# Debiasing the Conversion Rate Prediction Model in the Presence of Delayed Implicit Feedback

**DOI:** 10.3390/e26090792

**Published:** 2024-09-15

**Authors:** Taojun Hu, Xiao-Hua Zhou

**Affiliations:** 1 Department of Biostatistics, School of Public Health, Peking University, Beijing 100191, China; hutaojun@stu.pku.edu.cn; 2 Beijing International Center for Mathematical Research, Peking University, Beijing 100871, China

**Keywords:** recommender system, implicit feedback, delayed feedback, conversion prediction, selection bias

## Abstract

The recommender system (RS) has been widely adopted in many applications, including online advertisements. Predicting the conversion rate (CVR) can help in evaluating the effects of advertisements on users and capturing users’ features, playing an important role in RS. In real-world scenarios, implicit rather than explicit feedback data are more abundant. Thus, directly training the RS with collected data may lead to suboptimal performance due to selection bias inherited from the nature of implicit feedback. Methods such as reweighting have been proposed to tackle selection bias; however, these methods omit delayed feedback, which often occurs due to limited observation times. We propose a novel likelihood approach combining the assumed parametric model for delayed feedback and the reweighting method to address selection bias. Specifically, the proposed methods minimize the likelihood-based loss using the multi-task learning method. The proposed methods are evaluated on the real-world Coat and Yahoo datasets. The proposed methods improve the AUC by 5.7% on Coat and 3.7% on Yahoo compared with the best baseline models. The proposed methods successfully debias the CVR prediction model in the presence of delayed implicit feedback.

## 1. Introduction

The recommender system (RS) is widely used to address the information overload problem in multiple fields, including online advertising [1], drug adverse event prediction [2], and personalized treatments [3]. Predicting the conversion rate (CVR) plays a central role in RS. Taking online advertising as an example, advertisers are interested in evaluating the purchase potential of each item for the users; in this example, the CVR of a user–item pair refers to the probability of the user purchasing the item. By accurately predicting the CVR, advertisers can grasp users’ preferences and provide personalized advertisements to the users. On top of this, advertisers can raise their advertisement earnings with limited advertisement investment by enhancing the overall CVR. In general, a good CVR prediction model presents advantages in inferring user preference and offering personalized recommendations, improving the usefulness of the RS.

In practice, high-quality explicit ratings or feedback may be lacking. Instead, there is a large amount of implicit feedback, which indirectly reflects opinions by observing user behaviors [4]. One of the most severe problems of implicit feedback is the selection bias due to the nature of implicit feedback [5]. By passively tracing users’ past behaviors, the RS cannot collect user behaviors if the item is not exposed to the user. The collection of observed user–item pairs with implicit feedback is often a set of missing-not-at-random samples for the population [5]. Thus, directly inferring user preference from the observed behavior may lead to suboptimal performance of the CVR prediction model.

The CVR prediction problem with implicit feedback has been studied for a long time [6,7,8,9]. To address the selection bias of implicit feedback, content-based approaches collect and utilize the features of users and items along with contextual information. This information helps to mine the users’ actual preferences by creating a profile of each user and item, then matching the user–item pairs by associating their profiles. In addition, methods incorporating propensity scores can help to alleviate the selection bias, including the inverse probability score weighting (IPS) and double robust (DR) methods. These methods have been applied to several popular models dealing with implicit feedback, including matrix factorization (MF) [10] and neural collaborative filtering (NCF) [11], resulting in a large number of approaches for tackling selection bias and predicting CVR using implicit feedback [12,13,14,15,16,17].

However, previous methods omit the delayed feedback problem, which often occurs in implicit feedback scenarios due to limited observation times. For example, if a user likes an item, the user will convert at a certain post-click timestamp. If the timestamp is outside the observation period, then negative feedback will be observed, resulting in a false negative sample. As pointed out by [18], delayed feedback is very common in practice, where most conversions occur after a certain period; 50% of conversions occur after one day post-click, and 13% of those occur after two weeks. Ignoring delayed feedback in predicting CVR leads to underestimation of the eventual CVR.

There have been a number of methods aiming to adjust for the naïve method of capturing the delayed feedback. Most of these tackle delayed feedback issues by assuming that a fixed proportion of the negative label will finally convert and that every user–item pair without conversions shares the same probability as a mislabel. However, this is not reliable, as it has been shown that the delayed time is not a uniform distribution, with most conversions occurring within the latest day after the click. To this end, Chapelle et al. [18] first introduced the survival analysis method to deal with delayed feedback in CVR prediction. This method assumes that the delay from click to conversion follows an exponential distribution with unknown expectations. The paper stresses that the models of delayed feedback and CVR prediction cannot be taken separately. The CVR prediction model was trained by maximizing the joint likelihood of both models with either the EM algorithm or direct optimization. Following this work, several authors have extended the exponential delayed feedback model to a more flexible Weibull model/mixture of Weibull model or even the nonparametric model, borrowing ideas from the good performance of kernel density estimation [19,20,21].

The above methods either deal with the selection bias brought about by the implicit feedback or the delayed feedback issues with explicit feedback separately; however, no methods have tackled the delayed feedback when only the implicit feedback is available for predicting the CVR. Disentangling these two problems in predicting CVR and ignorance of either side would lead to a biased CVR prediction. In this paper, we propose methods for unbiased CVR prediction in the presence of delayed implicit feedback. We apply two methods that have been validated as useful in debiasing RS in the context of implicit feedback, namely, the IPS and DR methods, and consider how to obtain unbiased CVR predictions with delayed feedback in these methods. We propose the joint likelihood of observed data in conversion logs with parameters in the CVR prediction and delayed feedback models. In parameter estimation, we minimize the joint loss function, that is, the negative log-likelihood with regularization. Most existing methods either address the selection bias using basic IPS or DR techniques or their variants, or capture the delayed feedback while assuming no selection bias. Compared with existing methods, our proposed joint likelihood loss and multi-task learning approach seeks to simultaneously optimize the CVR prediction model and the delayed feedback model; thus, the proposed models should be unbiased and more robust. We carried out experiments with the proposed method on two real-world datasets. The results demonstrate good performance in obtaining a higher CVR prediction accuracy with unbiased samples from the test datasets. The proposed approach outperforms competing models, including those proposed to address selection bias with implicit feedback and those dealing with delayed feedback, on multiple measurement scales.

## 2. Related Work

### 2.1. Conversion Rate (CVR) Prediction

Conversion rate prediction refers to estimating the probability of a user completing a predefined action (a conversion), such as making reviews/ratings for a movie, making a purchase, or signing up for a service in e-commerce. Fundamental models such as logistic regression and multi-layer perception can be used for prediction by leveraging some features from the users and items. More sophisticated methods such as the ESMM [22], ESCM2 [23], and ECMA [24], have been proposed and applied in different industries. However, these methods ignore the existence of delayed feedback in practice. ESCD [25] and ESDF [26] tackle the issue of delayed feedback for their CVR prediction models. Nonetheless, these approaches need more theoretical assurance. In [23], the authors proved that the results of ESMM [22] were biased. In this paper, we propose a likelihood-based loss function and train the proposed models by maximizing the likelihood. The theoretical properties of maximized likelihood estimators help to enhance the evidence of the proposed method.

### 2.2. Selection Bias in Implicit Feedback

Implicit feedback refers to indirect indicators of users’ preferences, as opposed of explicit ratings; examples include browsing data and purchase history. Explicit feedback such as ratings explicitly reflects user preferences. Unlike explicit feedback, which requires a user response, implicit feedback is often readily available. However, implicit feedback has several drawbacks that challenge researchers to make full use of it, such as inherent noise and lack of negative feedback [27]. Several techniques have been proposed to handle these challenges, including negative sampling and methods for denoising [28,29,30]. However, the above methods have not addressed the issue of selection bias in implicit feedback. Thus, we apply the IPS approach to adjust for the selection bias. The DR approach can further improve models for CVR prediction by introducing an imputation model. The DR approach avoids the out-of-bounds problem and reduces the variance compared with the IPS approach. A number of of methods for debiasing RS have been proposed based on IPS and DR [12,14,31,32,33,34].

### 2.3. Delayed Feedback

Delayed feedback refers to situations where there is time between the click and the conversion. Ignoring the possibility of delayed feedback and directly labeling such samples as negative leads to underestimation of the CVR. As the delay time can be treated as a random variable following specific distributions of a survival time, it links CVR prediction with survival analysis in statistics. Delayed feedback models (DFM) [18] address this problem by assuming the distribution of the delayed time as an exponential distribution with an expectation determined by a generalized linear model having some predefined features. In addition, other distributions might be effective for modeling the delayed feedback. In [19,20], the authors proposed modeling the delayed feedback using a single Weibull distribution and a mixture of Weibull distributions, respectively, while [21] proposed an efficient approach called NoDeF for modeling the distribution of the delayed feedback nonparametrically using kernel density estimation techniques. However, the above methods may fail to reflect the true distribution of the delayed feedback. To resolve this problem, FSIW [35], FNW/FNC [36], ESDFM [37], DEFER [38], and DEFUSE [39] utilize resampling. However, all of the above methods rely on explicit feedback. Our proposal aims to provide more unbiased and accurate CVR prediction models in the presence of delayed implicit feedback.

## 3. Problem Setup and Preliminaries

### 3.1. Problem Setup

We first show the basic setups in a CVR prediction problem without considering the time delay from click to conversion in the presence of implicit feedback. Let U and I be the sets of users and items in the RS, respectively. The user–item pairs are expressed by D={(u,i)∣u∈U,i∈I}. We denote the conversion of user–item pair (u,i) by $r_{u,i}$. Conversions in the presence of implicit feedback often denote a variable that measures the user’s preference, such as whether the user views or purchases the item, rather than a direct rating or feedback to declare their like and dislike of the item(s). Without loss of generality, the conversion is assumed to be a binary variable, where $r_{u,i}=1$ means conversion and $r_{u,i}=0$ indicates a failure of conversion. In addition to the conversion outcomes, we denote the user–item features and the observation of $r_{u,i}$ by $x_{u,i}\in R^{K}$ and $o_{u,i}\in \left\{0,1\right\}$ for each user–item pair in the dataset, where $o_{u,i}=1$ indicates that $r_{u,i}$ is observed and documented in the database. We denote the sets of observed user–item pairs in the dataset by $O=\{\left(u,i\right)|\left(u,i\right)\in D,o_{u,i}=1\}$. The complete observations can be represented by $\left\{x_{u,i},r_{u,i}|\left(u,i\right)\in O\right\}\cup \left\{x_{u,i}|\left(u,i\right)\in D\setminus O\right\}$.

We assume a model for CVR prediction with parameters θ which seeks to predict $r_{u,i}$ with user–item features $x_{u,i}$, which we denote by ${\hat{r}}_{u,i}=f\left(x_{u,i},\theta \right)$. The aim is to achieve the least biased CVR prediction for all user–item pairs among the population D. To this end, by assuming a prediction error $err_{u,i}=L\left(f\left(x_{u,i},\theta \right),r_{u,i}\right)$, e.g., the mean square or cross-entropy error, the problem translates to minimizing the population loss, that is,

$$L_{p}\left(\theta \right)=\frac{1}{\left|D\right|}\sum_{(u,i)\in D}err_{u,i}=\frac{1}{\left|D\right|}\sum_{(u,i)\in D}L\left(f\left(x_{u,i},\theta \right),r_{u,i}\right).$$

However, there are also unobserved user–item pairs (u,i)∈D∖O. The naïve method optimizes the loss function on observed samples, which seeks to minimize the sample loss:

(1) $$L_{s}\left(\theta \right)=\frac{1}{\left|O\right|}\sum_{(u,i)\in O}err_{u,i}=\frac{1}{\left|O\right|}\sum_{(u,i)\in O}L\left(f\left(x_{u,i},\theta \right),r_{u,i}\right).$$

If the set of observed samples O is a random selection from the population D without selection bias, in the other words, the selection indicator $o_{u,i}$ is independent with $\left(x_{u,i},r_{u,i}\right)$, then we have $E\left(L_{s}\left(\theta \right)\right)=E\left(L_{p}\left(\theta \right)\right)$; thus, minimizing the sample loss can asymptotically achieve the optimal estimation based on the population loss, leading to the unbiased CVR prediction model. However, due to the properties of implicit feedback there might exist a selection bias which renders $o_{u,i}$ dependent with $\left(x_{u,i},r_{u,i}\right)$ [14]. There have been a number of methods addressing this issue. The IPS method addresses this issue using the propensity score, that is, the probability of each user–item pair being observed, then weights the sample loss using the inverse propensity scores. The loss function of the IPS method is

(2) $$L_{IPS}\left(\theta \right)=\frac{1}{\left|O\right|}\sum_{(u,i)\in O}\frac{o_{u,i}err_{u,i}}{{\hat{p}}_{u,i}},$$

where ${\hat{p}}_{u,i}$ is the estimated propensity score of user–item pair (u,i). The IPS method reweighs the observed user–item pairs using the inverse propensity score. It has been verified that the IPS loss is unbiased of the population loss if the propensity score model is correctly specified, where ${\hat{p}}_{u,i}=E\left(o_{u,i}=1|x_{u,i}\right)$ for all (u,i)∈D. The DR method aims to improve the IPS method by incorporating an imputation model $g(x_{u,i},\phi )$ [14]. The loss function for the DR method is

(3) $$L_{DR}\left(\theta ,\phi \right)=\frac{1}{\left|O\right|}\sum_{(u,i)\in O}\left[\frac{o_{u,i}err_{u,i}}{{\hat{p}}_{u,i}}+\left(1-\frac{o_{u,i}}{{\hat{p}}_{u,i}}\right){\hat{err}}_{u,i}\right],$$

where ${\hat{p}}_{u,i}$ is the estimated propensity score and ${\hat{err}}_{u,i}=L\left(g\left(x_{u,i},\phi \right),r_{u,i}\right)$ is the loss of the imputation model. It has been proven that the DR method can avoid out-of-bounds prediction for the CVR and reduce the variance compared with the IPS method with the help of the auxiliary imputation model.

### 3.2. Delayed Feedback and Survival Analysis

In the presence of delayed feedback, the naïve method mistakenly assigns negative labels to those samples with observation times that are too short to capture the eventual conversion status. Previous studies [18] have pointed out that this issue is closely related to censored time survival analysis. In survival analysis, researchers are interested in estimating the occurrence of a predefined event, such as the resurgence of the disease after a treatment and the incubation time before symptoms after an infection. To this end, estimation of the period during the starting time (time of treatment or infections) and the event time, called the survival time, is of great importance. The length of the survival time is always larger than zero; thus, an ordinary normal distribution cannot be applied, as some survival time may be missing when the actual survival time is longer than the observation time. This issue makes survival analysis different from general regression or classification problems.

In survival analysis, assuming the survival time T(T>0) follows a distribution with the probability density function f(t), the cumulative distribution function is denoted by $F\left(t\right)=\int_{0}^{t}f\left(s\right)ds$. The survival function is the probability of the survival time being longer than the time *t*, in other words, where the event does not occur before time *t*. The survival function is denoted by $S\left(t\right)=\mathrm{Pr}\left(T>t\right)=\int_{t}^{+\infty }f\left(s\right)ds=1-F\left(t\right)$. We denote the observation time (or censored time in traditional survival analysis) by *C*. The probability that the event does not occur before the observation time is S(C). The hazard function λ(t), referring to the instantaneous occurrence rate of the event happening in time *t* conditional on the absence of occurrence before time *t*, plays an important role in survival analysis. It can be computed using λ(t)=f(t)/S(t). The functions mentioned above can all be expressed as a function of λ(t), that is,

(4) $$\begin{array}{cc} S(t) & =\exp(-\int_{0}^{t}\lambda \left(s\right)ds),\\ F(t) & =1-S\left(t\right)=1-\exp(\int_{0}^{t}-\lambda \left(s\right)ds),\\ f(t) & =\lambda \left(t\right)S\left(t\right)=\lambda \left(t\right)\exp(\int_{0}^{t}-\lambda \left(s\right)ds). \end{array}$$

By designing λ(t), it is possible to consider multiple types of survival time distributions, including exponential, gamma, Weibull, log-normal, etc. The simplest is the exponential distribution, where λ(t)=λ is a constant. Though simple, the exponential distribution has strong ability to fit the real-world survival time. Here, we apply the simplest exponential distribution for delayed feedback modeling and show its advantage in CVR prediction with implicit and delayed feedback without the need for a sophisticated delayed feedback model.

## 4. Materials and Methods

The goal of the proposed method is to predict the CVR of a user–item pair given its features. We denote this by the prediction model $f(x_{u,i},\theta )$. Overall, we realize this goal by assuming a parametric model for the delayed feedback and maximizing a newly proposed likelihood function given the parametric model and all the observed data. Directly maximizing the likelihood (1) can lead to biased estimates for the parameters and a biased CVR prediction model due to selection bias and delayed feedback. We revise the likelihood function (1) to tackle these two problems simultaneously. First, we assume an exponential model for the delayed feedback and use it to infer the probability of a conversation being observed/unobserved given its real status. In comparison, the naïve approach assumes that the observed status of the conversions implies the actual status for all user–item pairs, which is impossible in the presence of delayed feedback. Second, we incorporate an IPS/DR module to tackle the selection bias, which makes the estimated parameters robust against selection bias. We optimize the modified likelihood with multi-task learning to obtain a more unbiased CVR prediction model.

In the following, we use uppercase letters for random variables and lowercase letters for observed data. The variables in the model are provided below (note that the subscripts (u,i) are omitted in some cases for simplification):

- X: Set of features, including the features of the user and those of the item.
- O∈{0,1}: Random variable indicating whether the user–item pair is observed in the dataset.
- C∈{0,1}: Random variable indicating whether a conversion will finally happen.
- D≥0: Delay between click and conversion.
- E≥0: Observation time (elapsed time) after the click.
- R∈{0,1}: Variable indicating whether the conversion occurs during the observation time.

As an example, O(u,i) represents the random variables indicating whether user–item pair (u,i) is observed, and $o_{u,i}$ represents the realization of O(u,i) in the dataset.

In addition, there is a period between the item being displayed and the click. Mostly, the click occurs just a few seconds after the display, and is captured by the observation time between the display and the click; thus, we regard it as a momentary event. The connection between these variables plays a key role in describing the model. If a conversion finally occurs, it suggests that the conversion will finally happen, and the delay time between the click and conversion is captured by the observation time, that is,

(5) R=1⟺C=1,D≤E.

If a conversion has not been observed, this may be attributed to two possible cases: either a conversion will not finally happen, or the observation time is too short to capture the delayed feedback, that is,

(6) R=0⟺C=0orC=1,D>E.

We can observe *Y* if and only if O=1; thus, some $r_{u,i}=R\left(u,i\right)$ would be lacking. We assume independence between *O* and (C,D,E) given the features *X*, that is,

(7) Pr(C,D,E∣X,O=1)=Pr(C,D,E∣X).

This assumption corresponds to the ignorability assumption under the causal inference [40], and is often assumed to guarantee identifiability. We also assume independence of the pair (C,D) and the observation time *E* given the set of features *X*, i.e.,

(8) Pr(C,D∣X,E)=Pr(C,D∣X).

This assumption holds, as the analysis system decides the observation time irrespective of the nature of the users or items; in contrast, the random variables (C,D) are determined by the intrinsic nature of the user–item interactions.

From the datasets, the conversions, features, and observation times for the observed user–item pairs are given. Following the previous section, we denote the whole set of user–item pairs by D and the set of observed user–item pairs by O. The observed datasets consist of triplets $\left(x_{u,i},r_{u,i},e_{u,i}\right)$ for (u,i)∈O. As for those with $r_{u,i}=1$, the delay time from click to conversion $d_{u,i}$ is given, forming an alternative triplet $\left(x_{u,i},r_{u,i},d_{u,i}\right)$. To fit the data, we first derive the probability of $R\left(u,i\right)=r_{u,i}=1,D\left(u,i\right)=d_{u,i}$ and $R\left(u,i\right)=r_{u,i}=0,E\left(u,i\right)=e_{u,i}$ given $X\left(u,i\right)=x_{u,i}$ and the parameters of the models, which are the foundation of the overall likelihood function. First, according to (5), we have

(9) $$\begin{array}{cc} \; & \mathrm{Pr}(r_{u,i}=1,D\left(u,i\right)=d_{u,i}|X\left(u,i\right)=x_{u,i})\\ = & \mathrm{Pr}(D\left(u,i\right)=d_{u,i},C\left(u,i\right)=1|X\left(u,i\right)=x_{u,i})\\ = & \mathrm{Pr}\left(D\left(u,i\right)=d_{u,i}|X\left(u,i\right)=x_{u,i},C\left(u,i\right)=1\right)\mathrm{Pr}\left(C\left(u,i\right)=1|X=x_{u,i}\right), \end{array}$$

and according to (6),

(10) $$\begin{array}{cc} \; & \mathrm{Pr}(r_{u,i}=0|X\left(u,i\right)=x_{u,i},E\left(u,i\right)=e_{u,i})\\ = & \mathrm{Pr}\left(D\left(u,i\right)>e_{u,i},C\left(u,i\right)=1|X\left(u,i\right)=x_{u,i},E=e_{u,i}\right)+\mathrm{Pr}\left(C\left(u,i\right)=0|X\left(u,i\right)=x_{u,i},E\left(u,i\right)=e_{u,i}\right). \end{array}$$

We use two models to fit the observed data, including models for $\mathrm{Pr}(D\left(u,i\right)=d_{u,i}|X\left(u,i\right)=x_{u,i},C\left(u,i\right)=1)$ and $\mathrm{Pr}(C\left(u,i\right)=1|X\left(u,i\right)=x_{u,i})$. The first model is the distribution of the delayed time and the latter is the CVR prediction model, which is the main focus of our problem. We assume the hazard function as λ(x,t)(t≥0). By changing λ(x,t), it is possible to obtain multiple parametric models for the delayed feedback, e.g., Weibull distribution, gamma distribution, log-normal distribution, log-logistic distribution, etc. The most common is the exponential distribution, with λ(x,t)=λ(x) as time-invariant. Without loss of generality, we assume the delayed feedback model as the simplest exponential distribution. Thus, according to (4),

(11) $$\mathrm{Pr}\left(D\left(u,i\right)=d_{u,i}|X\left(u,i\right)=x_{u,i},C\left(u,i\right)=1\right)=\lambda \left(x_{u,i}\right)\exp\left(-\lambda \left(x_{u,i}\right)d_{u,i}\right)$$

and

(12) $$\mathrm{Pr}\left(D\left(u,i\right)>e_{u,i}|X\left(u,i\right)=x_{u,i},C\left(u,i\right)=1\right)=S\left(x_{u,i},e_{u,i}\right)=\exp\left(-\lambda \left(x_{u,i}\right)e_{u,i}\right).$$

We model the connection of the hazard function $\lambda (x_{u,i})$ with a modified linear regression, that is, $\lambda \left(x_{u,i}\right)=\mathrm{Max}\left(0,\beta \cdot x_{u,i}\right)$, where β is an unknown parameter to be estimated. On constructing the CVR prediction model $\mathrm{Pr}(C\left(u,i\right)=1|X\left(u,i\right)=x_{u,i})$, we use matrix factorization, which assumes a latent embedding of users, denoted by $H_{u}$ for user u∈U and $W_{i}$ for item *i*. The probability of conversion for user–item pair (u,i) is denoted by ${\hat{r}}_{u,i}=1/\left[1+\exp\left(-H_{u}\cdot W_{i}\right)\right]$. Then, we have

(13) $$\begin{array}{cc} \mathrm{Pr}(C\left(u,i\right)=1|X\left(u,i\right)=x_{u,i}) & ={\hat{r}}_{u,i},\\ \mathrm{Pr}(C\left(u,i\right)=0|X\left(u,i\right)=x_{u,i}) & =1-{\hat{r}}_{u,i}. \end{array}$$

According to Equations (9) to (13), the loss function of a single user–item pair (u,i) is provided by the modified cross-entropy loss, which is also the negative log-likelihood function:

(14) $$\begin{array}{cc} err_{u,i} & =r_{u,i}\log\left[\mathrm{Pr}\left(r_{u,i}=1,D\left(u,i\right)=d_{u,i}|X\left(u,i\right)=x_{u,i}\right)\right]\\ & +\left(1-r_{u,i}\right)\log\left[\mathrm{Pr}\left(r_{u,i}=0|X\left(u,i\right)=x_{u,i},E\left(u,i\right)=e_{u,i}\right)\right]\\ & =r_{u,i}\log\left[{\hat{r}}_{u,i}\lambda \left(x_{u,i}\right)\exp\left(-\lambda \left(x_{u,i}d_{u,i}\right)\right)\right]+\left(1-r_{u,i}\right)\log\left[{\hat{r}}_{u,i}\exp\left(-\lambda \left(x_{u,i}e_{u,i}\right)\right)+1-{\hat{r}}_{u,i}\right]. \end{array}$$

We propose two loss functions based on the IPS and DR approaches. For the one incorporating the IPS approach, we obtain the joint loss function $L_{IPS}\left(\theta ,\beta \right)=\frac{1}{\left|O\right|}\sum_{(u,i)\in O}\frac{o_{u,i}err_{u,i}}{{\hat{p}}_{u,i}}$, where $err_{u,i}$ refers to (14). The parameters are optimized by minimizing the regularized loss function

(15) $$\arg\min_{\theta ,\beta }L_{IPS}\left(\theta ,\beta \right)+\frac{\gamma }{2}{∥\theta ∥}_{2}^{2}+\frac{\mu }{2}{∥\beta ∥}_{2}^{2},$$

where (γ,μ) are regularization parameters. We call this method **MF-IPS+DeF**.

For the loss function incorporating the DR approach, we assume another imputation model, which is also encoded with an MF denoted by ${\hat{rr}}_{u,i}=g\left(x_{u,i},\phi \right)$. Thus, ${\hat{err}}_{u,i}$ is provided by the binary cross-entropy loss, which is also the negative log-likelihood function:

(16) $${\hat{err}}_{u,i}={\hat{rr}}_{u,i}\log\left({\hat{r}}_{u,i}\right)+\left(1-{\hat{rr}}_{u,i}\right)\log\left(1-{\hat{r}}_{u,i}\right).$$

We then obtain the joint loss $L_{DR}\left(\theta ,\beta ,\phi \right)=\frac{1}{\left|O\right|}\sum_{(u,i)\in O}\left[\frac{o_{u,i}err_{u,i}}{{\hat{p}}_{u,i}}+\left(1-\frac{o_{u,i}}{{\hat{p}}_{u,i}}\right){\hat{err}}_{u,i}\right]$, where $err_{u,i}$ refers to (14) and ${\hat{err}}_{u,i}$ refers to (16). The parameters are obtained by minimizing the loss with regularization:

(17) $$\arg\min_{\theta ,\beta ,\phi }L_{DR}\left(\theta ,\beta ,\phi \right)+\frac{\gamma }{2}{∥\theta ∥}_{2}^{2}+\frac{\mu }{2}{∥\beta ∥}_{2}^{2}+\frac{\tau }{2}{∥\phi ∥}_{2}^{2},$$

where (γ,μ,τ) are regularization parameters. We jointly learn the parameters in the CVR prediction model and the imputation model using the multi-task learning technique. We call this method **MF-DR-JL+DeF**.

## 5. Results

This section presents the results of our experiments comparing the proposed methods and competing methods for predicting the CVR on two real-world datasets.

### 5.1. Dataset Preparation

Following previous studies [14,33,34], we conducted real-world experiments on the widely used Coat [12] and Yahoo [41] benchmark datasets. Both datasets consist of training and test sets, with the two sets in each study sharing the same sets of users and items. The training datasets are biased datasets that suffer from selection bias, while the test datasets are unbiased. The Coat dataset consists of 6960 user–item pairs in the training set and 4640 user–item pairs in the test set, with 290 users and 300 items; each user–item pair is represented by a rating from 1 to 5. We binarized these using a threshold of 3, with ratings lower than 3 assigned a score of zero 0 and a score of 1 being assigned otherwise. The Yahoo dataset consists of 311,704 user–item pairs in the training set and 54,000 in the test set. We binarized these similarly, again using a five-point scale.

We simulated the delayed feedback as follows: first, we extracted the features of users and items through a basic MF method applied to the training set. We concatenated the features of users and items for the representation of the features of user–item pairs $x_{u,i}$, then fixed the overall training period *L* as a constant. The user may randomly click an item at a timestamp $t_{click}$ within the training period. We sampled $t_{u,i}^{click}$ from a uniform distribution Unif(0,L), while the observation time (or elapsed time) was $E_{u,i}=L-t_{u,i}^{click}$. We assumed that the delayed time followed an exponential distribution $D_{u,i}∼\mathrm{Exp}\left(\lambda \left(x_{u,i}\right)\right)$, where the hazard function $\lambda \left(x_{u,i}\right)=\exp\left(W_{d}\cdot x_{u,i}\right)$, $W_{d}$ is randomly sampled from a prior multivariate normal distribution $N(0_{p},\sigma_{H}^{2}I_{p})$, and $\sigma_{H}$ is a hyperparameter. In the presence of delayed feedback, the user–item pairs with $D_{u,i}>E_{u,i}$ might be mistakenly labeled as negative samples. We set the labels of these samples to 0 in the training set, and set L=5 and $\sigma_{H}=0.1$ in the basic setups, rendering around 20% of positive samples in the training set mislabeled as 0 due to delayed feedback. We trained the proposed method and competing methods on the modified training set, then tested them on the unbiased test set.

### 5.2. Training Details

For the proposed **MF-IPS+DeF** and **MF-DR-JL+DeF** methods, we minimized the loss function with the Adam optimizer. We tuned the learning rate in {0.01,0.015,0.02,0.03,0.05,0.1} and weight decay in $\left[1\times 10^{-6},5\times 10^{-2}\right]$. The training was stopped if there were five epochs with a relative loss reduction lower than $1\times 10^{-5}$. In the proposal with DR, we set different weight decay rates in the CVR prediction model/imputation model and the delayed feedback model, then tuned them separately. The batch size was set to 128 for the Coat dataset and 256 for the Yahoo dataset. All the results and figures were generated with Python codes. We trained the models with Python 3.11.9 and Pytorch 2.3.0. The figures were generated with matplotlib 3.8.4.

### 5.3. Baselines

We want to show the performance of the proposed methods in the presence of both implicit and delayed feedback; however, the baseline methods for comparison include methods that address selection bias through the IPS approach, which follow the DR approach while ignoring the delayed feedback, and those that capturing the delayed feedback with parametric models, which neglect the selection bias. As our proposed proposed CVR prediction model addresses delayed feedback using MF as the base model, we compare it with other MF-based methods such as MF [10], MF-IPS [12], MF-ASIPS [31], MF-SNIPS [12], and MF-DR [31], MF-DR-JL [14], and MF-MRDR-JL [32]. MF-IPS, MF-ASIPS, and MF-SNIPS are variants of the base MF method with base or advanced IPS approaches, while MF-DR, MF-DR-JL, and MF-MRDR-JL are variants of the base MF method that incorporate DR techniques; all of these methods overlook the issue of delayed feedback. We compared the proposed approach with the delayed feedback models, assuming different distributions for the delayed time. We used the method in [18], called the DFM, as the base model. In [18], the authors proposed considering the delayed time as an exponential distribution and used a multi-layer perception (MLP) to learn the hazard function. They used the Weibull and log-normal distributions, which are popular in survival analysis, to model the delayed time. To distinguish them, they referred to these as DFM-EXP, DFM-Weibull, and DFM-LN. All three of these methods are designed to capture the delayed feedback, but neglect the impact of selection bias in the datasets.

### 5.4. Evaluation Protocols

We adopted three widely used metrics for evaluation: AUC, NDCG@K, and Recall@K. AUC stands for area under the receiver operating curve, which is a widely used metric in diagnostic studies to evaluate the accuracy of a diagnostic test by synthesizing the pairs of sensitivity and specificity across all cutoff points of the continuous biomarker of the test. NDCG@K stands for the normalized discounted cumulative gain at K, which compares the top K predicted items for each user and the actual preference of each user. Recall@K aggregates and averages the proportion of correctly specified items among the top K predicted items for each user. Both NDCG@K and Recall@K are useful for comparing the ranking performance of different ranking algorithms. We set K=10 for Coat and K=5 for Yahoo. Each model was trained for ten rounds, retrieving the mean and standard error as the eventual results.

### 5.5. Performance Comparisons

The prediction performance of the proposals and the baselines for the two datasets are shown in Table 1. Among the baselines, the DR-based methods perform better than the IPS-based and delayed feedback models. For the Coat dataset, the MF-DR model obtains the highest AUC. For the Yahoo dataset, the MF-MRDR-JL, which jointly learns both the imputation model and the prediction model, achieves the best AUC. Both use the DR approach to eliminate the selection bias, indicating the effectiveness of this approach. The DFM model, which incorporates the delayed feedback into CVR prediction, improves the AUC of MLP by around 0.02 and largely enhances NDCG@K and Recall@K, suggesting the importance of training a delayed feedback model and including it in the loss function. In contrast, the DFM-based methods, which consider the delayed feedback but ignore the selection bias, perform significantly worse than the SOTA MF-based baseline methods and the proposed methods, indicating that it is particularly important to adjust the selection bias when addressing CVR prediction with implicit feedback. The proposed approach significantly outperforms the state-of-the-art (SOTA) baselines on all evaluation metrics. The proposed **MF-DR-JL+DeF** method improves upon the AUC of the MF-DR method by 5.7% on the Coat dataset, and the proposed **MF-IPS+DeF** method improves upon the AUC of the MF-MRDR-JL method by 3.7% on the Yahoo dataset. While the MF-IPS method performs significantly worse than the SOTA baselines, the proposed **MF-IPS+DeF** method greatly improves upon its the performance. From this perspective, the delayed feedback module plays an important role in accurate CVR prediction; indeed, both the delayed feedback module and adjusting for selection bias are indispensable.

### 5.6. Sensitivity Analysis

**Figure 1:**
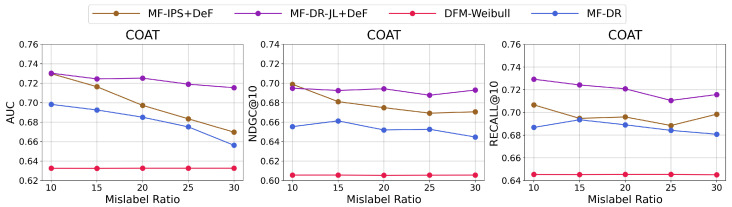
Effects of the mislabeling ratio (%) on AUC, NDGC@10, and Recall@10 on the Coat dataset.

To comprehensively evaluate the proposed method, we conducted a sensitivity analysis with the proposed **MF-IPS+DeF** and **MF-DR-JL+DeF** methods on the Coat dataset and compared them with two baselines: the optimal DFM model (DFM-Weibull) and the optimal MF-based method (MF-DR). First, we varied the mislabeling ratio (MR), that is, the proportion of positive samples mislabeled as negative with observation times shorter than the delay time, from 10% to 30%. We set different MRs by changing the total observation time *L*; L=3.2,3.92,5,6.7,9.7 correspond to MR = 10.02%,15.05%,19.66%,24.99%,30.12%, respectively, or approximately 10%, 15%, 20%, 25%, and 30%. The results are shown in Figure 1. The proposed methods consistently outperform the baselines in terms of AUC, NDGC@K, and Recall@K. Compared with the other two methods, **MF-DR-JL+DeF** shows a comparably small decrement on all metrics when the MR increases, suggesting its robustness. The DFM-Weibull method consistently performs the worst, and there is no significant variation in its performance across different MRs. This showcases the importance of adjusting for selection bias in CVR prediction with implicit feedback.

**Figure 2:**
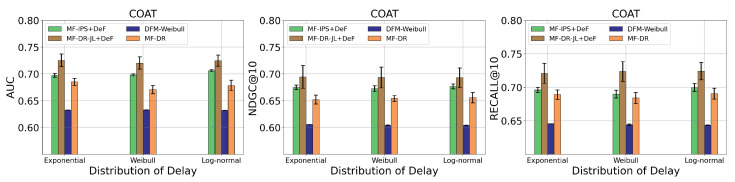
Effects of different distributions of the delay time in terms of AUC, NDGC@10, and Recall@10 on the Coat dataset.

We additionally explored the effect of different distributions of the delayed feedback on the performance of the proposed methods, as shown in Figure 2. In addition to the exponential distribution, we set two other distributions (the Weibull and log-normal distributions) for the delay time. The MR was fixed to around 20%. Note that even for the other distributions, the proposed **MF-IPS+DeF** and **MF-DR-JL+DeF** methods only adopted the exponential distribution when constructing the loss function. The performance of the proposed methods under the different distribution shows no significant differences, with both methods consistently outperforming the baselines. This suggests the robustness of the proposals under mis-specification of the delay time distribution.

### 5.7. Comments

From the results of the performance comparisons under the original setups and the sensitivity analysis, different parametric distributions of the delayed feedback have little impact on the predicted CVR. Thus, we recommend using the easiest exponential model for the delayed feedback. The proposed **MF-DR-JL+DeF** method is robust against an increasing mislabeling ratio caused by delayed feedback, while the other methods may not be as robust. In practice, we recommend the **MF-DR-JL+DeF** method for an unbiased and robust CVR prediction model in the presence of delayed and implicit feedback.

## 6. Discussion

We experimented with the proposed methods on two real-world datasets and compared the results with extensive baselines. Of all the baseline methods, the ones with the optimal AUCs were all DR-based methods. Compared with the IPS approach, DR methods incorporate an imputation model to reduce the variance. Of the three baseline delayed feedback models, there were no significant differences in prediction performance. A possible reason for this result is that the actual distribution of the delay time is generated from the simplest exponential distribution. Thus, delayed feedback models with more complex Weibull and log-normal distributions also capture the actual exponential distribution of the delay time. Therefore, we conducted a sensitivity analysis, varying the actual underlying distribution of the delayed feedback. The results show that the performance of these methods is consistent across different actual delayed feedback distributions. Thus, we recommend using the simplest exponential distribution to model the delayed feedback.

We only used MF as the base model in the proposed method. In addition to the basic MF model, other methods such as BPR and NCF [11,42] can be applied to implicit feedback problems. These baseline models can be easily combined with the proposed method by adjusting the loss functions, and may further improve the CVR prediction accuracy. In addition, we only tried two approaches for tackling selection bias with implicit feedback: the basic IPS method, and the DR method with joint learning. There are several more recent methods studying eliminating the selection bias based on the basic IPS and DR methods, such as by balancing the covariates or proposing better propensity score estimators [34,43,44]. The proposed method can be extended to incorporate these methods for further improvement.

There are several limitations in the proposed method that must be addressed. First, the proposed method relies on the joint loss, which requires an explicit form of the distribution for the delayed feedback. The proposed models only use the exponential distribution, and show good performance even when the actual distribution is a Weibull or log-normal distribution. Nevertheless, the distribution of the delayed feedback may be complex and have multiple peaks or periodicity [21]. For example, users may view the purchase web pages and complete conversions in a certain period during the day, called the peak user hours. To this end, several methods have been proposed to capture the complex distribution of delayed feedback [35,36,37,38,39]. We intend to extend our proposed method in future work to more accurately model the delayed feedback. Second, we only considered static CVR prediction in the problem setups. Other topics that have raised attention in recent times include multi-touch attribution settings and streaming CVR prediction [18,19,37,45,46]. In further work, it would be appealing to test the usefulness of the proposed method or its extensions for addressing these problems.

## 7. Conclusions

In this paper, we have proposed a novel method for debiasing CVR prediction in RS in the presence of delayed and implicit feedback. We tackle selection bias and mislabeling due to delayed feedback by devising a new likelihood-based loss function that jointly incorporates models for the delayed time and debiasing the CVR prediction through the IPS technique and DR method. We conducted extensive experiments on two real-world datasets, showing that the proposed methods can obtain state-of-the-art performance in the presence of implicit and delayed feedback. The results of our sensitivity analysis validate the robustness of the proposed **MF-DR-JL+DeF** model, which we recommend for practical use.

## Figures and Tables

**Table 1:** Performance in terms of AUC, NDCG@*K*, and Recall@*K* on Coat and Yahoo. The best two results are bolded; the best baseline results for DFM methods with delay feedback and IPS/DR-based methods are underlined.

	Coat			Yahoo		
**Method**	**AUC**	**NDCG@10**	**RECALL@10**	**AUC**	**NDCG@5**	**RECALL@5**
MLP	0.617±0.001	0.505±0.000	0.551±0.001	0.573±0.006	0.569±0.009	0.336±0.009
DFM-EXP	0.632±0.000	0.605±0.000	0.645±0.000	0.598±0.001	**0.645** ±0.001	**0.413** ±0.001
DFM-Weibull	0.633±0.000	0.605±0.000	0.645±0.000	0.598±0.001	0.645±0.001	0.413±0.002
DFM-LN	0.631±0.000	0.605±0.000	0.644±0.000	0.598±0.001	0.645±0.001	0.413±0.002
MF	0.652±0.009	0.650±0.007	0.688±0.007	0.500±0.002	0.598±0.002	0.366±0.002
+ IPS	0.642±0.010	0.640±0.005	0.684±0.010	0.609±0.002	0.601±0.003	0.382±0.004
+ ASIPS	0.656±0.008	0.646±0.010	0.685±0.011	0.606±0.003	0.618±0.004	0.400±0.004
+ SNIPS	0.650±0.006	0.652±0.008	0.691±0.006	0.563±0.004	0.588±0.004	0.355±0.005
+ DR	0.685 ±0.007	0.652±0.008	0.689±0.007	0.604±0.004	0.626±0.003	0.399±0.003
+ DR-JL	0.658±0.009	0.656±0.008	0.690±0.007	0.627±0.002	0.601±0.003	0.381±0.004
+ MRDR-JL	0.678±0.007	0.666 ±0.008	0.692 ±0.008	0.629 ±0.003	0.611±0.004	0.398±0.005
+ **IPS+DeF**	**0.698** ±0.004	**0.674** ±0.006	**0.695** ±0.008	**0.652** ±0.002	0.600±0.002	0.373±0.004
+ **DR-JL+DeF**	**0.724** ±0.010	**0.695** ±0.021	**0.721** ±0.015	**0.644** ±0.013	**0.665** ±0.020	**0.432** ±0.018

## Data Availability

The original data and codes presented in the study are openly available at https://github.com/Taojun-Hu/MF-DeF, accessed on 30 July 2024.
